# Development of an Impedimetric Aptasensor for Label Free Detection of Patulin in Apple Juice

**DOI:** 10.3390/molecules24061017

**Published:** 2019-03-13

**Authors:** Reem Khan, Sondes Ben Aissa, Tauqir A. Sherazi, Gaelle Catanante, Akhtar Hayat, Jean Louis Marty

**Affiliations:** 1 BAE: Biocapteurs-Analyses-Environnement, Universite de Perpignan Via Domitia, 52 Avenue Paul Alduy, 66860 Perpignan CEDEX, France; Kreemjadoon@gmail.com (R.K.); sondes.benaissa@fst.utm.tn (S.B.A.); gaelle.catanante@univ-perp.fr (G.C.); 2 Department of chemistry, COMSATS University Islamabad, Abbottabad Campus, Abbottabad 22060, Pakistan; sherazi@cuiatd.edu.pk; 3 Interdisciplinary Research Centre in Biomedical Materials (IRCBM), COMSATS University Islamabad, Lahore Campus, Lahore 54000, Pakistan; 4 Université de Tunis El Manar, Faculté des Sciences de Tunis, Laboratoire de Chimie Analytique et Electrochimie (LR99ES15), Sensors and Biosensors Group, Campus Universitaire de Tunis El Manar, Tunis 2092, Tunisia

**Keywords:** impedimetric aptasensor, screen-printed interface, bifunctional polymer arms, PAT detection, apple juice

## Abstract

In the present work, an aptasensing platform was developed for the detection of a carcinogenic mycotoxin termed patulin (PAT) using a label-free approach. The detection was mainly based on a specific interaction of an aptamer immobilized on carbon-based electrode. A long linear spacer of carboxy-amine polyethylene glycol chain (PEG) was chemically grafted on screen-printed carbon electrodes (SPCEs) via diazonium salt in the aptasensor design. The NH_2_-modified aptamer was then attached covalently to carboxylic acid groups of previously immobilized bifunctional PEG to build a diblock macromolecule. The immobilized diblocked molecules resulted in the formation of long tunnels on a carbon interface, while the aptamer was assumed as the gate of these tunnels. Upon target analyte binding, the gates were assumed to be closed due to conformational changes in the structure of the aptamer, increasing the resistance to the charge transfer. This increase in resistance was measured by electrochemical impedance spectroscopy, the main analytical technique for the quantitative detection of PAT. Encouragingly, a good linear range between 1 and 25 ng was obtained. The limit of detection and limit of quantification was 2.8 ng L^−1^ and 4.0 ng L^−1^, respectively. Selectivity of the aptasensor was confirmed with mycotoxins commonly occurring in food. The developed apta-assay was also applied to a real sample, i.e., fresh apple juice spiked with PAT, and toxin recovery up to 99% was observed. The results obtained validated the suitability and selectivity of the developed apta-assay for the identification and quantification of PAT in real food samples.

## 1. Introduction

Effective detection of low molecular weight toxic molecules is vital in areas such as environmental monitoring and the food industry [1,2,3]. Mycotoxins are small agro-based food contaminants that pose serious health threats to humans and animals [4]. Patulin (PAT) is a low molecular weight mycotoxin produced by various fungal species. This small mycotoxin is commonly present in fruit- and vegetable-based products, especially apples [5,6]. The antibiotic properties of PAT were firstly reported in 1940. Some studies have also shown the adverse health effects of PAT on higher plants and animals [7]. Extensive studies have been carried out to highlight the health risks of PAT, and it has been concluded that PAT exhibits several chronic, acute, and cellular level toxic effects both on human and animals [8]. PAT has neurotoxic, immunosuppressive, and mutagenic effects on several animal species and cause severe damage to the intestinal epithelium, which leads to degeneration, inflammation, ulceration, and bleeding [8,9]. Due to such pronounced toxicities, different authorities have set regulatory limits for PAT in food. The European Union (EU) is the first organization that defined a maximum admissible limit, i.e., 50 μg L^−1^ [10].

To monitor the level of PAT in food, several analytical techniques have been established including HPLC, thin layer chromatography with mass spectrometry and UV detection [11], gas chromatography coupled with mass spectrometry [12], micellar electrokinetic chromatography (MEKC), and colorimetry [13,14,15,16]. Despite their high reliability and accuracy, these techniques suffer many disadvantages such as complex instrumentation, high running cost, and complicated handling methods that make on-site analysis unsuitable. Owing to such drawbacks, there is an immense need of reliable, fast, and easy-to-operate screening methods.

Biosensors, being simple, reliable, and fast screening tools, have emerged as an attractive alternative to classical analytical methods. Among different types of biosensors, electrochemical aptasensors offer significant advantages over the others. [17]. Change in the unique three-dimensional structure of aptamers upon target binding provides great flexibility in developing electrochemical aptasensors. In most cases, an aptamer is tagged with a redox label such as ferrocene or methylene blue [18]. These types of biosensor designs have been successfully employed to detect different targets, including mycotoxins. However, their main problem is that they require large conformational changes in the aptamer structure to tune the distance of the electro-active label from the transducer surface. Moreover, the process of labeling aptamers is expensive, and labeling sometimes decreases the activity of the modified biomolecules, which results in a more complex, laborious, and time-consuming assay design. [19]. As an alternative, interest in the development of simpler and cheaper label-free aptasensors has recently increased. These platforms are generally based on electrochemical impedance spectroscopy (EIS) as a powerful technique, allowing sensitive detection of the smallest variations in electron transfer processes. Still, in the case of small analytes, target binding events do generate an easily measurable impedimetric signal, because physical hindrance induced by these low molecular weight analytes is often insufficient to create detectable variation. However, ultrasensitive detection of low weight targeted analytes by a simple aptasensor design is quite difficult. Different methods have been used for signal amplification, such as strand displacement amplification, rolling circle amplification, and the use of nanomaterials [20,21]. Although these approaches offer advantages in signal intensification, they are costly, are complex, and have difficult operating conditions. These problems have resulted in the exploration of novel aptasensor designs to ensure the sensitive detection of low molecular weight targets. Integration of a spacer in aptasensor design could be a simple and effective method of signal amplification. In this work, we present this strategy for the detection of a small mycotoxin, i.e., PAT.

Furthermore, considering the mycotoxin analysis, multiple efforts have been made to design and develop biosensors for aflatoxins and ochratoxins, but very few reports are available for PAT detection [22,23]. In the present work, a novel impedimetric aptasensor was designed for the label-free detection of PAT. A long spacer, i.e., carboxy-amine PEG, was used to facilitate the label-free detection and enhance the sensitivity of the developed aptasensor. Disposable screen-printed carbon electrodes (SPCEs) were used as transducers. The SPCEs were based on a conventional three-electrode system with a working electrode made up of graphite (with a circular disk 4 mm in diameter), a counter electrode (curved line: 16 × 1.5 mm), and Ag/AgCl (straight line: 16 × 1.5 mm) as a pseudo reference electrode. All parameters were carefully optimized. The developed aptasensor was then assessed for a real sample, i.e., apple juice. To the best of our knowledge, no such impedimetric aptasensor has yet been reported for the detection of PAT.

## 2. Materials and Methods 

### 2.1. Design and Working Principle of Impedimetric Aptasensor 

**Scheme 1:**
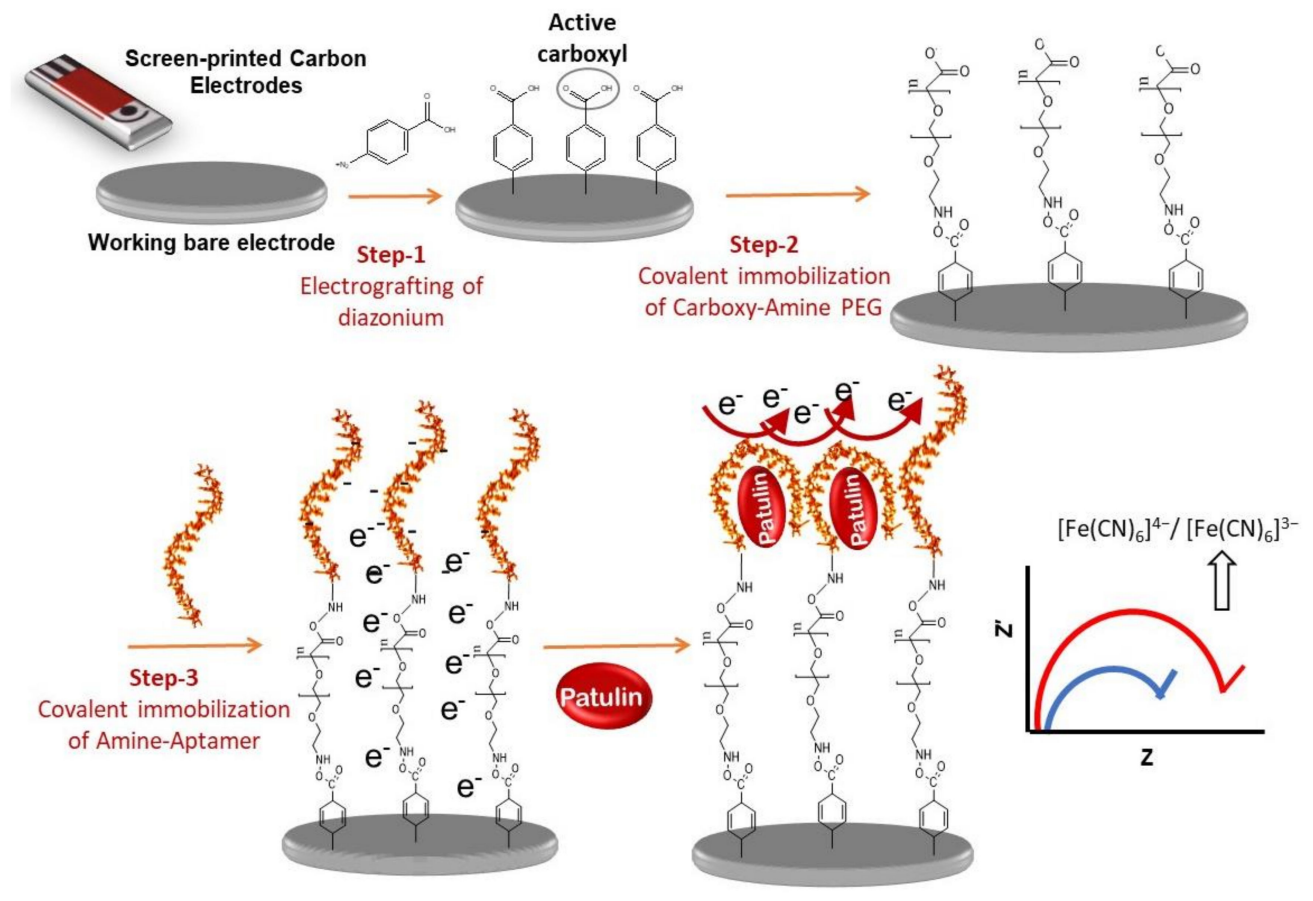
Design and working principle of the proposed aptasensor.

A schematic representation of the working principle and the stepwise design strategy of the developed impedimetric aptasensor for PAT detection is shown in Scheme 1. As indicated, three major steps were carried out for the fabrication process. In the proposed aptasensor, a diblock macromolecule (carboxy-amine PEG + anti-PAT-aptamer) was chemically grafted onto the surface of SPCEs to form clusters of long spacer arms on the working electrode. Aminobenzoic acid was first electrochemically grafted onto the surface of SPCEs through linear sweep voltammetry (LSV). This technique is based on the electrochemical reduction of diazonium salt, leading to aryl-centered radicals by the spontaneous release of nitrogen. The voltammogram in Figure S1 shows the successful reduction of diazonium cations via a one-electron process. An insulating layer of carboxyphenyl was covalently formed on the surface.

Diazonium deposition was then followed by the chemical grafting of carboxy-amine PEG through the amide bond. The long spacer arms of heterobifunctional PEG acted like tunnels for the electrons of the redox marker to reach the surface of the electrode. The second part of the diblock, i.e., the anti-PAT aptamer, acted as gates for these tunnels. In the absence of a targeted analyte, the aptamer remained unfolded, and it is assumed that the gates were opened, thereby permitting the electron transfer from the redox probe to the surface of the electrode. Meanwhile, in the presence of the targeted analyte, 3D conformational changes in the aptamer structure resulted in the formation of an aptamer-PAT quadruplex complex that locked the gates of tunnels, resulting in blockage of the electron flow toward the electrode surface [24]. Furthermore, the conformational changes in the aptamer structure also resulted in the exposition of the negatively charged backbone of the aptamer. Consequently, steric hindrance after the formation of the quadruplex complex and the electrostatic repulsion between the anionic ferri/ferrocyanide redox system and the negatively charged aptamer collectively increased the impedance of the system. The higher the concentration of PAT is, the higher the impedance will be. This concept makes the quantitative detection of PAT feasible.

### 2.2. Electrochemical Characterization of the Aptasensors 

**Figure 1:**
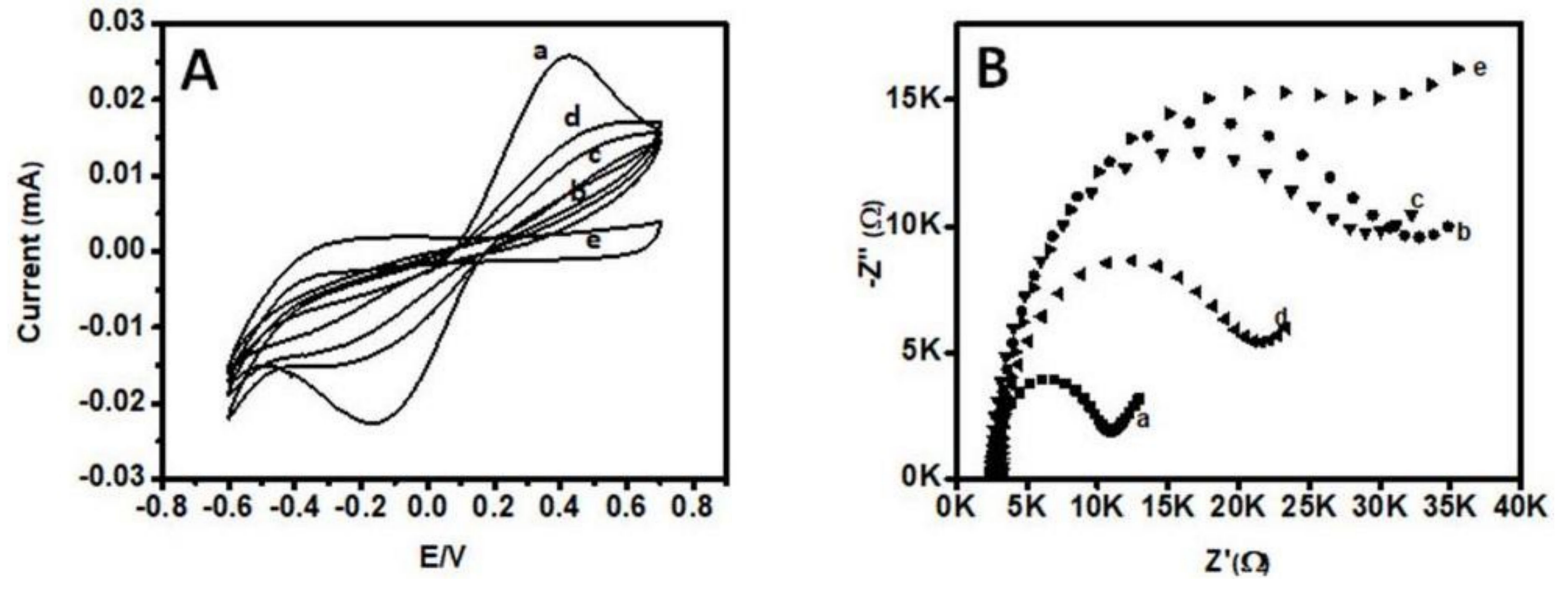
Characterization of modification steps during aptasensor fabrication. (**A**) Cyclic voltammogram obtained in 1 mM Ferri/Ferrocyanide after each modification step during aptasensor fabrication: (a) bare SPCE; (b) carboxyphenyl modified SPCE; (c) after immobilization of carboxy-amine PEG; (d) anti-patulin (anti-PAT) aptamer modified electrode; (e) after incubation of 50 ng L^−1^ patulin (PAT). (**B**) Nyquist plots of a 1 mM [Fe(CN)_6_]^3−/4−^ probe in PBS, pH 7.4.

Cyclic voltammetry (CV) and EIS are widely used techniques to characterize the electrode/electrolyte interfacial properties at different modification steps of aptasensor fabrication. Thus, the different preparation stages were investigated by recording the impedance spectra and cyclic voltammograms of the modified electrode in the presence of the reversible [Fe (CN)_6_]^4−/3−^ redox system, shown in Figure 1. 

#### 2.2.1. Cyclic Voltammetry Characterization

CV in the ferri/ferrocyanide solution is a useful tool for examining the electrochemical behavior of functionalized electrodes, as electron transfer is more or less feasible depending on the hindrance of steric barriers. Therefore, this technique was used to investigate and characterize the electrode surface modifications. Figure 1A shows the cyclic voltammograms obtained after each modification of the electrode surface during aptasensor fabrication. Bare SPCEs exhibited a symmetric oxidation and reduction peaks (Figure 1A(a)), but the insulating layer of 4-aminobenzoic acid (ABA) blocked electron transfer from the probe to the electrode surface, so no oxidation or reduction took place, as we can see in Figure 1A(b). After that, bifunctional PEG was deposited through the amide bond. The long linear spacer arms of carboxy-amine PEG allow the electron to move from the probe solution to the electrode surface. Hence, an increase in redox peaks of cyclic voltammogram can be observed in Figure 1A(c). The immobilization of the aptamer over carboxy-amine PEG further decreased the resistance of the system and facilitated the charge transfer from the solution to the electrode surface, resulting in an increase in the redox shape of the voltammogram (Figure 1A(d)). After the completion of aptasensor fabrication, PAT was incubated over the aptasensor. Changes in the 3D conformation of the aptamer structure offered steric hindrance and resulted in the exposition of the negative charges of the aptamer that strongly repelled the redox probe. No redox reaction occurred at the electrode surface, as shown in Figure 1A(e). 

#### 2.2.2. Impedance Characterizations

EIS is a powerful and a useful tool for depicting the barrier properties of the modified electrode. The R_et_ values obtained after fitting the curves to a Randles equivalent circuit are shown in Table S1 for various stages of aptasensor fabrication. Faradic impedance spectra presented in the form of Nyquist plots are shown in Figure 1B for the stepwise modification process. Bare SPCEs exhibited a small semicircle, as shown in Figure 1B(a), indicating a very low resistance in electron transfer from the redox probe to the electrode surface. After the electro-grafting of ABA via the diazonium ion, the resistance of the surface significantly increased (Figure 1B(b)). This increase in resistance was due to the presence of the COO^-^ surface, which acted as a barrier layer and hindered the electron transfer from the anionic ferri/ferrocyanide redox probe to the electrode due to strong electrostatic repulsion. The second modification was the covalent attachment of carboxy-amine PEG. For this purpose, the COOH group was first activated with EDC-NHS chemistry. After the activation of COOH carboxy-amine PEG was covalently attached via amide coupling between the terminal NH_2_ group of carboxy-amine PEG and the COO^-^ group of the carboxyphenyl layer, there was a significant increase in R_et_ (Figure 1B(c)). Contrary to the expected results, a small decrease in impedance was observed, as compared to that obtained after ABA electrodeposition, although in both cases COO^-^ was exposed on the surface. This comparative decrease in R_et_ can be attributed to the formation of tunnels on the electrode surface after the deposition of heterobifunctional PEG, which facilitates the electron flow from the probe solution to the electrode. In the case of a carboxyphenyl layer, these tunnels were absent, so the substrate was completely and uniformly covered with negative charges. The electrostatic repulsion between COO^-^ did not allow any electrons to transfer from the probe solution to the electrode. Such behavior of modified electrodes demonstrates that the formation of tunnels on the electrode surface is due to the long linear spacer arms of carboxy-amine PEG [24]. After successful immobilization of bifunctional PEG, the exposed COOH group was again activated by EDC-NHS. Subsequently, amine-terminated anti-PAT aptamer was immobilized via amide bond formation. A decrease in R_et_ in Figure 1B(d), as compared to 1B(c), proved the effective immobilization of the aptamer, as the electrostatic repulsion between COO^-^ and the redox probe was reduced after aptamer immobilization. The incubation of PAT increased in R_et_ values, which can be seen in Figure 1B(e). The quadruplex formed upon target recognition, blocked the molecular gates and exposed the negatively charged backbone of the aptamer toward the anionic probe that in-turn increased the impedance of system.

### 2.3. Optimization of Experimental Parameters

**Figure 2:**
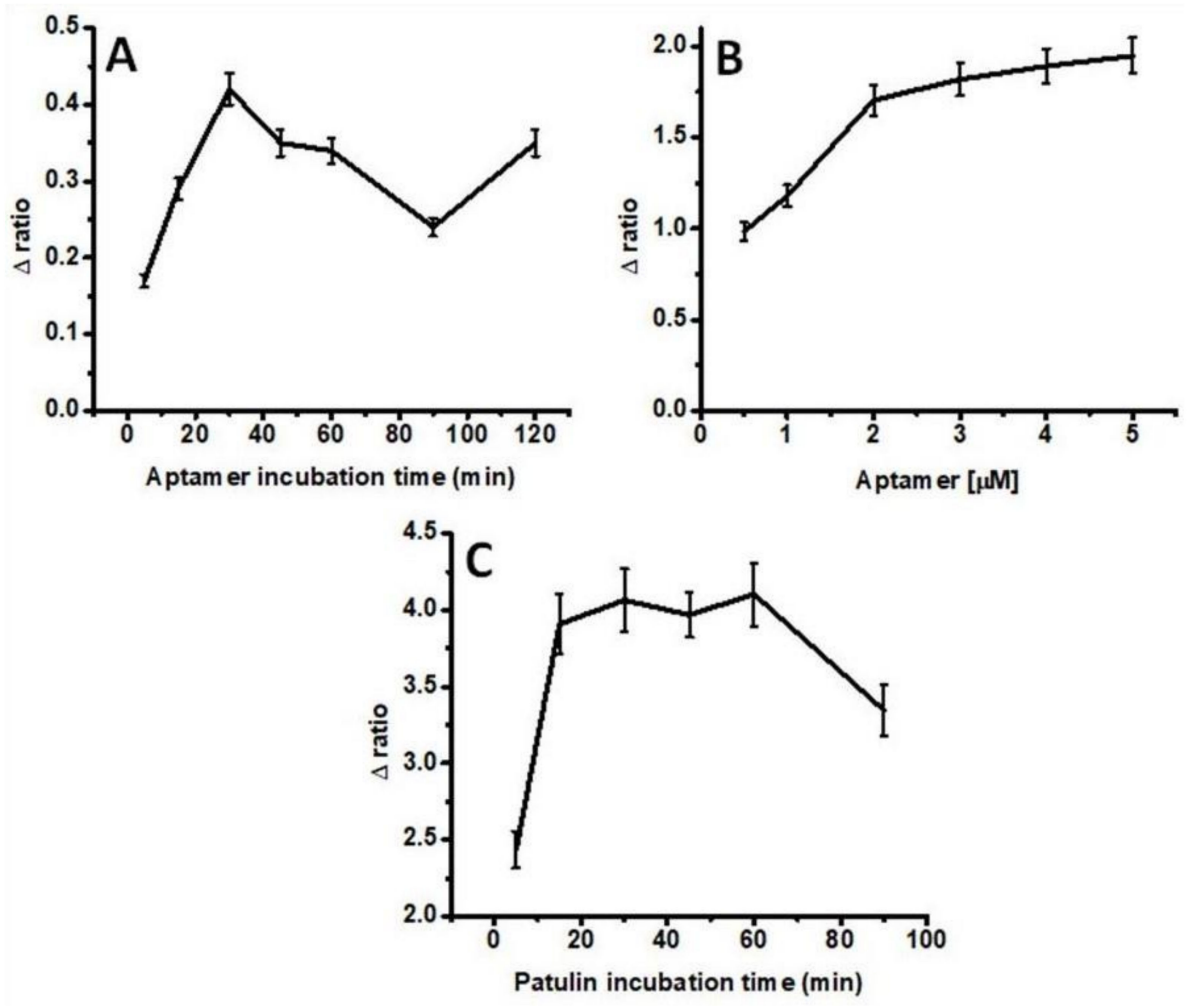
Optimization of experimental parameters. (**A**) Variation in Δ_ratio_ with increasing time after incubation with aptamer solution. (**B**) Variation in Δ_ratio_ with varying aptamer concentration. (**C**) Variation in Δ_ratio_ obtained at different intervals of time after the incubation of PAT.

The factors that can affect the electrochemical properties of the proposed aptasensor are pH, temperature, analyte incubation time, and the concentration of the aptamer immobilized on the electrode surface. Therefore, these parameters were optimized to obtain better analytical performance. Results are shown in Figure 2. The optimal response of the aptasensor was obtained at room temperature and pH 7.4. The concentration of carboxy-amine PEG was optimized to be 6 mg mL^−1^.

Furthermore, the time required to immobilize the maximum concentration of aptamers on the active surface of the SPCEs was optimized. Variation in the Δ_ratio_ was recorded in the time range of 5–90 min. It was observed that maximum immobilization of the aptamer was achieved after 30 min (Figure 2A).

The concentration of the aptamer is another important parameter. We recorded the Δ_ratio_ for aptamer concentration ranging between 0.5 and 5.0 µM. Results in Figure 2B indicated that the maximum Δ_ratio_ was observed at a 2.0 µM concentration. Therefore, 2.0 µM was considered as the optimum concentration and was used in the following experiments.

The analyte incubation time is also an important parameter optimized during the aptasensor fabrication. Optimum incubation time for the analyte was found to be 45 min, as shown in Figure 2C.

### 2.4. Analytical Performance of the Aptasensors

**Figure 3:**
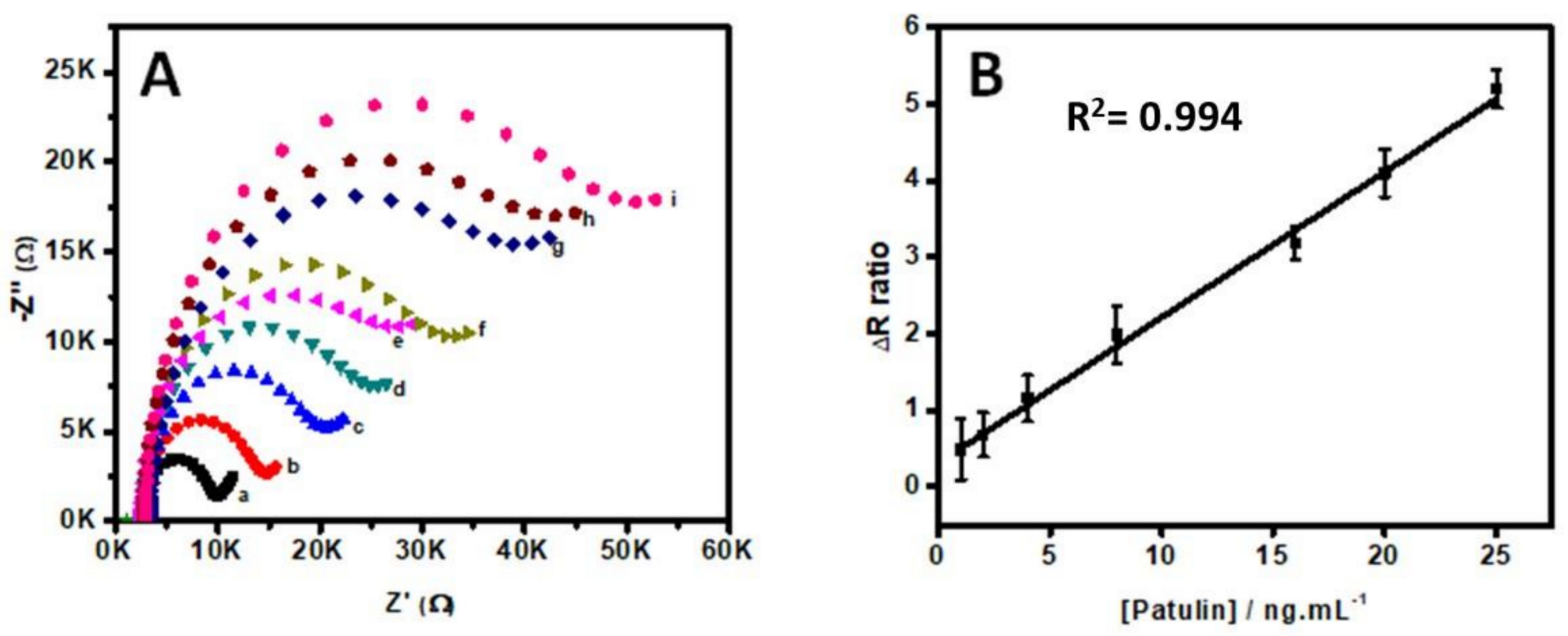
(**A**) Nyquist plot of the aptasensor after incubation with different PAT concentrations: (a) bare, (b) 1 ng mL^−1^, (c) 2 ngmL^−1^, (d) 4 ng mL^−1^, (e) 8 ng mL^−1^, (f) 16 ng mL^−1^, (g) 20 ng mL^−1^, (h) 25 ng mL^−1^, and (i) 50 ng mL^−1^. (**B**) Calibration plot of Δ_ratio_ with increasing PAT concentration.

To evaluate the analytical performance of the developed impedimetric aptasensor, different concentrations of PAT were incubated on the proposed aptasensing platform. A significant increase in the R_et_ was observed with increasing PAT concentration. A Nyquist plot for the electrochemical aptasensor in response to different concentrations of PAT is presented in Figure 3. The results indicated that the more PAT was bound to the electrode surface, the higher the R_et_ of the system was. This proportional increase in R_et_ indicated that the developed aptasensor can quantitatively detect PAT. In order to calculate a reliable estimation of increase or decrease in impedance, Δ_ratio_ was calculated for all aptasensors (different electrodes developed in triplicate). 

Figure 3B shows that the Δ_ratio_ of the aptasensor gradually increases with an increasing concentration of PAT. 

Limit of detection and limit of quantification were determined by calculating the signal-to-noise ratio in the blank. The formulae are

LOD = 3 * standard deviation of blank / S

LOQ = 10 * standard deviation of blank / S

where “S” is the slope of calibration curve.

The limit of detection obtained from the impedimetric aptasensor is 2.8 ng L^−1^, while the limit of quantification is 4.0 ng L^−1^. 

### 2.5. Selectivity of the Aptasensors 

**Figure 4:**
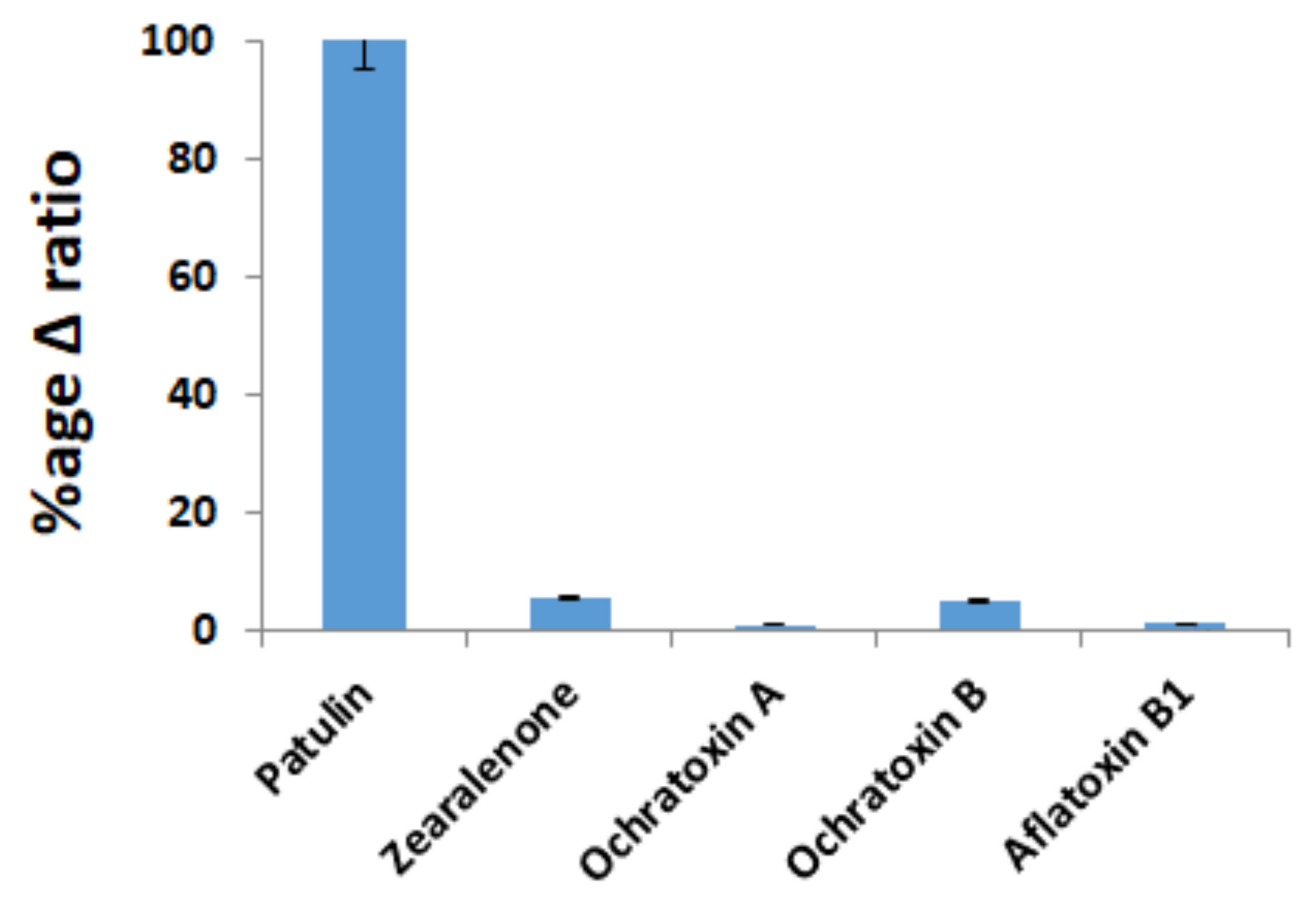
Selectivity test for the developed aptasensor.

Selectivity of the aptasensor against PAT was checked by incubating the aptasensor with different interfering analytes such as ochratoxin A, ochratoxin B, and aflatoxins. Obtained results are presented in Figure 4. No significant increase in R_et_ values can be seen in the case of mycotoxins other than PAT, which confirms the high selectivity and specificity of the aptasensor for PAT. In the same context, a control single-strand DNA was immobilized on the transducer surface in a fashion similar to that described for immobilization of the aptamer. No significant change in R_et_ was observed in the presence of PAT. These results also prove that there were no non-specific binding sites on the transducer surface.

### 2.6. Application to Spiked Apple Juice Sample

The developed impedimetric aptasensor was evaluated for real sample analysis. For a real sample application, PAT was detected in apple juice. Apple juice samples were spiked with three different concentrations of PAT, i.e., 4, 10, and 20 ng mL^−1^. Controlled experiments were performed by detecting the same concentrations of PAT in phosphate binding buffer. The electrochemical response in apple juice exhibits the same phenomenon as observed in buffer. Results are presented in Table 1. This experiment confirms the feasibility of the aptasensor for field applications. The recovery values were calculated based on the integration of the Δ_ratio_ value (electrochemical impedimetric response) of the spiked value in the equation of the calibration curve.

Y = 0.3128 + 0.1901 * X

The precision and accuracy of the proposed aptasensor was confirmed by interday and intraday assay analysis. A relative standard deviation (RSD) of 2.8–4.0% (*n* = 3) was calculated for interday analysis, which indicated very good reproducibility of the assay. Similarly, for intraday analysis, RSD was 1.34%, which confirmed that results are reproducible.

## 3. Experimental 

### 3.1. Chemicals 

Amine-terminated aptamer sequence for PAT was purchased from Microsynth (Balgach, Switzerland) The aptamer sequence was developed by Shijia Wu et al. by a graphene oxide-assisted SELEX (systematic evolution of ligands by exponential enrichment) process. Initially, eight aptamer sequences were shortlisted, and an aptamer named PAT-11 was then selected as the final recognition element based on its low K_d_ (dissociation constant) value, i.e., 21.83 nM, and the highest binding affinity and selectivity for PAT. The potential applicability of the selected aptamer sequence was further confirmed by developing an enzyme-chromogenic substrate system-based colorimetric aptasensor for the selective and sensitive detection of PAT in a real matrix [25]:

Sequence of PAT aptamer:

5′GGC CCG CCA ACC CGC ATC ATC TAC ACT GAT ATT TTA CCT T-NH_2_-3′.

The sequence of control aptamers was as follows:

(5′ GCA GTT GAT CCT TTG GAT ACC CTG G3′)-NH_2_.

PAT, sodium phosphate dibasic Na_2_HPO_4_, potassium chloride KCl, potassium phosphate monobasic KH_2_PO_4_, calcium chloride CaCl_2_, magnesium chloride MgCl_2_, 4-aminobenzoic acid, carboxy-amine PEG (3400) ethyl-dimethyl-amino-propyl carbodiimide, *N*-hydroxy succinimide and ferricyanide, ferrocyanide, sodium nitrite, and 4-aminobenzoic acid were obtained from Sigma-Aldrich (Lyon, France). Sterilized water was used for aptamer solution preparation, while distilled water was used throughout the experimental procedure for reagent preparation. For real sample analysis, fresh apple juice was bought from a supermarket in Perpignan, France. 

Home-made SPCEs were used for the electrochemical experiments. 

### 3.2. Instrumentation 

Pre-treatment of the aptamer (heating at 90 °C for 8 min followed by immediate cooling to 4 °C for 4 min then 15 min at RT) was done on thermocycler mastercycler (Eppendorf, Le pecq, France). All the electrochemical measurements were performed on an Electrochemical Workstation “Biologics” equipped with EC-Lab software.

Lyophilized aptamer was diluted in phosphate binding buffer (PBB 50 mM). PBB was composed of 2 mM KH_2_PO_4_, 8mM Na_2_HPO_4_, 135 mM KCl, 60 mM NaCl, and 5 mM MgCl_2_ in deionized water. The pH of the buffer was adjusted to 7.4.

### 3.3. Experimental Protocol

#### 3.3.1. Covalent Immobilization of Aptamer onto the SPCEs

Before use, all electrodes were electrochemically washed with washing buffer (0.5 M H_2_SO_4_ diluted in 0.1 M KCl) with 8–10 CV scans between 1 and −1 V at 100 mV.s^−1^

Diazonium salt was generated by mixing 2 mM ABA in 0.5 M HCl with 2 mM sodium nitrite. The solution was left to react for 5 min for maximum generation of diazonium salt. After 5 min, 150–200 μL of this salt solution was electrodeposited on SPCEs via LSV with a potential ranging between 0.6 and −0.8 V and a scan rate of 50 mV.s^−1^ [26]. Furthermore, the exposed COOH group of ABA was activated through EDC/NHS chemistry (by immersing the electrode for 60 min in solution of 100 mM EDC and 25 mM NHS solution in 100 mM MES buffer pH = 5.5). After activation of COOH, 6 mM carboxy-amine PEG solution was then incubated on a working electrode for 40 min to attach the spacer covalently with aminobenzoic acid via an amide bond. The terminal COOH group of carboxy amine PEG was again activated through carbodiimide chemistry. Afterward, 2 µM NH2-modified PAT-aptamer was cast (20 μL) on the activated surface of the working electrode for an optimized immobilization time of 30 min. The NH_2_ group of the aptamer made a covalent bond with a terminal COOH group of PEG. Finally, the working area was treated with 1% BSA solution to block non-reactive sites, therefore avoiding the possibility of non-specific adsorption. 

All the modification steps were characterized by CV and EIS. The CV was performed within a potential range from 0.7 to −0.6 V at a 50 mV.s^−1^ scan rate. EIS measurements were performed using 1 mM [Fe (CN)_6_]^3−/4−^ solution as a free redox probe containing 10 mM KCl. 

The prepared electrode was stored at 4 °C and employed as a ready-to-use device during the present work. The ready-to-use electrodes did not show any variation in the electrochemical response over the entire period, suggesting the stability of the aptasensor. 

#### 3.3.2. Detection of Patulin

A stock solution of PAT was prepared in acetonitrile, and further dilutions were prepared in PBB. For the detection of PAT, selected concentrations of PAT were incubated for 1 h with a fabricated aptasensor, followed by rinsing with binding buffer, and electrochemical measurements were then carried out.

#### 3.3.3. Apple Juice Sample Preparation

In a 5 mL falcon tube, apple juice was vortex-mixed with ethyl acetate solution (1:1) and centrifuged at 10m000 rpm for 5 min. The supernatant was dried in a nitrogen environment. Afterward, the obtained dry powder was dissolved in PBS buffer after filtering with a nylon syringe filter (0.22 mm). Different PAT concentrations (a 4, 10, and 20 ng mL^−1^ final concentration) were then added to 50 μL of the pre-treated apple juice samples in order to perform standard additions and recovery experiments. The pH of the apple juice sample was adjusted to 7.4.

### 3.4. Impedimetric Measurements

Impedance measurements were obtained at an applied potential of 100 mV (vs. Ag/AgCl reference electrode), within a frequency range from 10 KHz to 0.5 Hz, an AC amplitude of 10 mV, and a sampling rate of 50 points. All measurements were performed in PBB containing a redox probe (1:1 mixture of 2 mM K_3_[Fe(CN)_6_]/K_4_[Fe(CN)_6_]). Impedance data was registered after each modification step of electrode surface in the following order: (i) bare electrode; (ii) diazonium salt deposition; (iii) carboxy-amine PEG immobilization; (iv) aptamer immobilization steps; and (v) PAT detection. The impedance spectra were presented as Nyquist plots, (−Z_im_ vs. Z_re_) and fitted to the Randles equivalent circuit model, as shown in Figure S1, with EC Lab software. The parameter R_s,_ corresponds to the solution resistance. R_et_ is the resistance in electron transfer between the electrode surface and the redox marker, whereas C is the double-layer capacitance and W (Warburg parameter) is associated with the diffusion of the redox probe. For all fittings, performed on EC-Lab software, the chi-square goodness-of-fit test was carefully checked to verify calculations (with 80.73 at a 92% confidence level). In this work, we focused on the variation in electron transfer resistance (R_et_). In order to minimize the electrode to electrode variations and to obtain reproducible and independent results, relative and normalized signals were required. Thus, the Δ_ratio_ parameter was calculated for all the electrodes. Δ_ratio_ is the ratio of R_et_ of the bare aptasensor to the R_et_ of the aptasensor with a particular amount of analyte. The R_et_ of the bare aptasensor was subtracted from the final values to ignore the electrode-to-electrode variation.

Δ_ratio_ = R_et (after PAT)_ – R_et (bare)_ / R_et (before PAT)_ − R_et (bare)_

where R_et (after PAT)_ is the value obtained incubating the aptasensor with the PAT, R_et (before PAT)_ is the electron transfer resistance value of the bare electrode, and R_et (bare)_ is the resistance in electron transfer of the blank electrode and buffer.

## 4. Conclusions

In summary, we successfully developed a field-portable impedimetric aptasensor for selective, sensitive, and label-free detection of the carcinogenic mycotoxin known as PAT. Electrochemical impedimetric aptasensing for label-free detection of PAT was based on diazonium chemistry and bifunctional polyethylene glycol as spacer. The carboxy-amine PEG proved to be an effective spacer that resulted in tunnel formation for the electron transfer from the redox probe to the electrode surface. The LOD and LOQ obtained were 2.8 ng L^−1^ and 4.0 ng L^−1^, respectively. Real sample analysis and interference study confirms that the developed aptasensor has the potential for PAT detection in the real matrix.

## Figures and Tables

**Table 1:** Recovery percentages obtained with designed electrochemical aptasensor for PAT monitoring in apple juice.

PAT Added (ng/mL)	PAT Found (ng/mL)	RSD %	RE %	R %
4	3.7	3.7	7.5	92.5
10	9.6	4.1	4	96
20	18.8	4.8	6	94

RSD % = relative standard deviation percentage; RE % = relative error percentage; R % = recovery percentage.

## Supplementary Materials

The following are available online at https://www.mdpi.com/1420-3049/24/6/1017/s1. Figure S1: Linear sweep voltammogram for electrodeposition of the in situ generated 4-carboxyphenyl diazonium salt in the diazotization mixture at SPCE, Figure S2: Randles Circuit for fitting of Nyquist Plot, Table S1: R_et_ Values obtained after each modification step of Aptasensor fabrication Electrode.

## References

[1] Xia X., He Q., Dong Y., Deng R., Li J. (2018). Aptamer-based Homogeneous Analysis for Food Control. Curr. Anal. Chem..

[2] Patra J.K., Mahato D.K., Kumar P. (2019). Biosensor Technology—Advanced Scientific Tools, With Special Reference to Nanobiosensors and Plant- and Food-Based Biosensors. Nanomaterials in Plants, Algae and Microorganisms.

[3] Vasilescu A., Marty J.L. (2016). Electrochemical aptasensors for the assessment of food quality and safety. Trends Anal. Chem..

[4] Laganà A. (2017). Introduction to the Toxins Special Issue on LC-MS/MS Methods for Mycotoxin Analysis.

[5] Moss M. (2008). Fungi, quality and safety issues in fresh fruits and vegetables. J. Appl. Microbiol..

[6] Ioi J.D., Zhou T., Tsao R., Marcone M.F. (2017). Mitigation of patulin in fresh and processed foods and beverages. Toxins.

[7] Iwahashi Y., Hosoda H., Park J.-H., Lee J.-H., Suzuki Y., Kitagawa E., Murata S.M., Jwa N.-S., Gu M.-B., Iwahashi H. (2006). Mechanisms of patulin toxicity under conditions that inhibit yeast growth. J. Agric. Food Chem..

[8] Glaser N., Stopper H. (2012). Patulin: Mechanism of genotoxicity. Food Chem. Toxicol..

[9] Mahfoud R., Maresca M., Garmy N., Fantini J. (2002). The mycotoxin patulin alters the barrier function of the intestinal epithelium: Mechanism of action of the toxin and protective effects of glutathione. Toxicol. Appl. Pharmacol..

[10] Commission E. (2006). Commission Regulation (EC) No 1881/2006 of 19 December 2006 setting maximum levels for certain contaminants in foodstuffs. Off. J. Eur. Union.

[11] Zheng Y., Wang X., He S., Gao Z., Di Y., Lu K., Li K., Wang J. (2018). Aptamer-DNA Concatamer-Quantum Dots Based Electrochemical Biosensing Strategy for Green and Ultrasensitive Detection of Tumor Cells via Mercury-Free Anodic Stripping Voltammetry. Biosens. Bioelectron..

[12] Sadok I., Szmagara A., Staniszewska M.M. (2018). The validated and sensitive HPLC-DAD method for determination of patulin in strawberries. Food Chem..

[13] Shephard G.S., Leggott N.L. (2000). Chromatographic determination of the mycotoxin patulin in fruit and fruit juices. J. Chromatogr. A.

[14] Takino M., Daishima S., Nakahara T. (2003). Liquid chromatography/mass spectrometric determination of patulin in apple juice using atmospheric pressure photoionization. Rapid Commun. Mass Spectrom..

[15] Gimeno A. (1979). Thin layer chromatographic determination of aflatoxins, ochratoxins, sterigmatocystin, zearalenone, citrinin, T-2 toxin, diacetoxyscirpenol, penicillic acid, patulin, penitrem A. J. Assoc..

[16] Beltrán E., Ibáñez M., Sancho J.V., Hernández F. (2014). Determination of patulin in apple and derived products by UHPLC–MS/MS. Study of matrix effects with atmospheric pressure ionisation sources. Food Chem..

[17] Grabowska I., Sharma N., Vasilescu A., Iancu M., Badea G., Boukherroub R., Ogale S., Szunerits S. (2018). Electrochemical Aptamer-Based Biosensors for the Detection of Cardiac Biomarkers. ACS Omega.

[18] Goud K.Y., Hayat A., Catanante G., Satyanarayana M., Gobi K.V., Marty J.L. (2017). An electrochemical aptasensor based on functionalized graphene oxide assisted electrocatalytic signal amplification of methylene blue for aflatoxin B1 detection. Electrochim. Acta.

[19] Rhouati A., Catanante G., Nunes G., Hayat A., Marty J.-L. (2016). Label-free aptasensors for the detection of mycotoxins. Sensors.

[20] Wu L., Xiong E., Zhang X., Zhang X., Chen J. (2014). Nanomaterials as signal amplification elements in DNA-based electrochemical sensing. Nano Today.

[21] Huang L., Wu J., Zheng L., Qian H., Xue F., Wu Y., Pan D., Adeloju S.B., Chen W. (2013). Rolling chain amplification based signal-enhanced electrochemical aptasensor for ultrasensitive detection of ochratoxin A. Anal. Chem..

[22] He B., Dong X. (2018). Aptamer based voltammetric patulin assay based on the use of ZnO nanorods. Microchim. Acta.

[23] Wu Z., Xu E., Jin Z., Irudayaraj J. (2018). An ultrasensitive aptasensor based on fluorescent resonant energy transfer and exonuclease-assisted target recycling for patulin detection. Food Chem..

[24] Hayat A., Andreescu S., Marty J.-L. (2013). Design of PEG-aptamer two piece macromolecules as convenient and integrated sensing platform: Application to the label free detection of small size molecules. Biosens. Bioelectron..

[25] Wu S., Duan N., Zhang W., Zhao S., Wang Z. (2016). Screening and development of DNA aptamers as capture probes for colorimetric detection of patulin. Anal. Biochem..

[26] Mahouche-Chergui S., Gam-Derouich S., Mangeney C., Chehimi M.M. (2011). Aryl diazonium salts: A new class of coupling agents for bonding polymers, biomacromolecules and nanoparticles to surfaces. Chem. Soc. Rev..

