# Supplementary material for: Development of an Impedimetric Aptasensor for Label Free Detection of Patulin in Apple Juice
Source: Molecules. 2019 Mar 13;24(6):1017. doi: 10.3390/molecules24061017 (PMC6471267; doi:10.3390/molecules24061017)
Supplement: Supplementary file 1 [file molecules-24-01017-s001.pdf]

molecules

Supplementary Materials

Development of an Impedimetric Aptasensor for Label Free Detection of Patulin in Apple Juice

Reem Khan [1,2,3], Sondes Ben Aissa [1,4], Tauqir A. Sherazi [2], Gaelle Catanante [1], Akhtar Hayat [3,*], and Jean Louis Marty [1,*]

[1] BAE: Biocapteurs-Analyses-Environnement, Universite de Perpignan Via Domitia, 52 Avenue Paul Alduy, 66860 Perpignan CEDEX, France; Kreemjadoon@gmail.com (R.K.); sondes.benaissa@fst.utm.tn (S.B.A.); gaelle.catanante@univ-perp.fr (G.C.)

[2] Department of chemistry, COMSATS University Islamabad, Abbottabad Campus, 22060, Pakistan, sherazi@cuiatd.edu.pk

[3] Interdisciplinary Research Centre in Biomedical Materials (IRCBM), COMSATS University Islamabad, Lahore Campus, 54000, Pakistan.

[4] Université de Tunis El Manar, Faculté des Sciences de Tunis, Laboratoire de Chimie Analytique et Electrochimie (LR99ES15), Sensors and Biosensors Group, Campus Universitaire de Tunis El Manar, 2092, Tunis, Tunisia

* Correspondence: akhtarhayat@cuilahore.edu.pk (A.H.); jlmarty@univ-perp.fr (J.L.M.); Tel.: +923317648291 (A.H.); +33468662257 (J.L.M.)

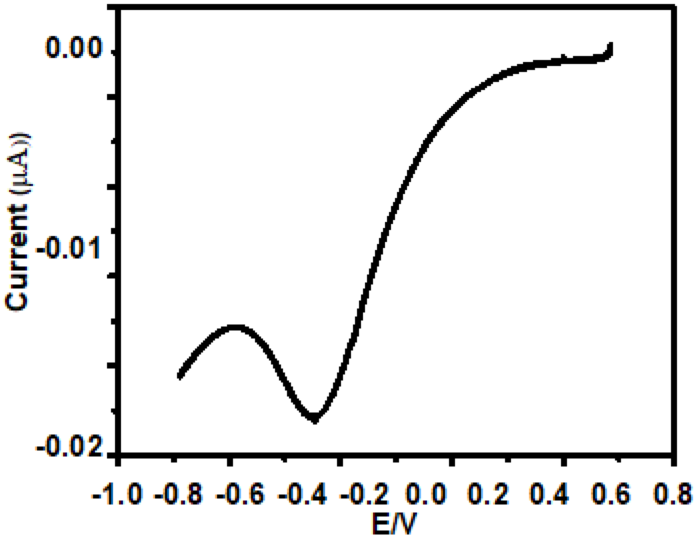

Figure S1. Linear sweep voltammogram for electrodeposition of the in situ generated 4-carboxyphenyl diazonium salt in the diazotization mixture at SPCE.

molecules MDPI

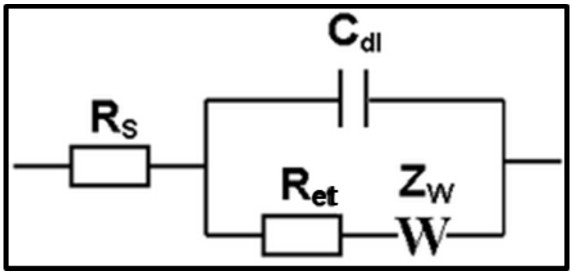

Figure S2. Randles Circuit for fitting of Nyquist Plot.

Table S1. R_{et} Values obtained after each modification step pf Aptasensor fabrication Electrode.

Different modification steps	R_{et} (KΩ)
Bare	7.56
After ABA	26.02
ABA+PEG	23.35
Aptasensor	16.10
Aptasensor + Patulin (50 ng mL^{-1})	24.76
